# Space Target Classification Improvement by Generating Micro-Doppler Signatures Considering Incident Angle

**DOI:** 10.3390/s22041653

**Published:** 2022-02-20

**Authors:** Jae-In Lee, Nammon Kim, Sawon Min, Jeongwoo Kim, Dae-Kyo Jeong, Dong-Wook Seo

**Affiliations:** 1 Interdisciplinary Major of Maritime AI Convergence, Korea Maritime & Ocean University, Busan 49112, Korea; rhadodehfdl@gmail.com; 2 Department of Land Radar, Hanwha Systems, Yongin 17121, Korea; nammoon.kim@hanwha.com (N.K.); junior.min@hanwha.com (S.M.); jeongwoo.kim@hanwha.com (J.K.); 3 Agency for Defense Development, Daejeon 34075, Korea; dkjeong@add.re.kr

**Keywords:** classification, convolution neural network, debris, deep learning, incident angle, micro-Doppler, space targets

## Abstract

Classifying space targets from debris is critical for radar resource management as well as rapid response during the mid-course phase of space target flight. Due to advances in deep learning techniques, various approaches have been studied to classify space targets by using micro-Doppler signatures. Previous studies have only used micro-Doppler signatures such as spectrogram and cadence velocity diagram (CVD), but in this paper, we propose a method to generate micro-Doppler signatures taking into account the relative incident angle that a radar can obtain during the target tracking process. The AlexNet and ResNet-18 networks, which are representative convolutional neural network architectures, are transfer-learned using two types of datasets constructed using the proposed and conventional signatures to classify six classes of space targets and a debris–cone, rounded cone, cone with empennages, cylinder, curved plate, and square plate. Among the proposed signatures, the spectrogram had lower classification accuracy than the conventional spectrogram, but the classification accuracy increased from 88.97% to 92.11% for CVD. Furthermore, when recalculated not with six classes but simply with only two classes of precessing space targets and tumbling debris, the proposed spectrogram and CVD show the classification accuracy of over 99.82% for both AlexNet and ResNet-18. Specially, for two classes, CVD provided results with higher accuracy than the spectrogram.

## 1. Introduction

**Figure 1:**
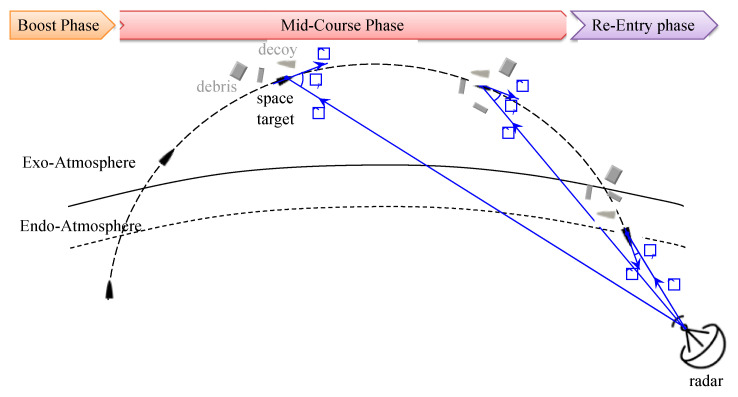
Flight phases of a ballistic missile and relative angle between the translation and radar line-of-sight.

The ballistic missile defense (BMD) system aims at detecting, tracking, classifying, and intercepting space targets. The flight of space targets is categorized into three phases–boost, midcourse, and re-entry phases, as shown in Figure 1. In the boost phase, a space target is launched and powered by rocket stages. For a BMD system, since the early detection and classification of the targets is important for rapid defense, it is preferable to detect a target in the boost phase rather than in other phases. In the boost phase, a high-resolution range profile (HRRP) is calculated from the radar received signal and the total length of the target is also estimated from the HRRP. The estimated total length can be used to classify space targets [1]. However, the low data update rate of 10 to 20 radar measurements in the boost phase makes it difficult to detect and classify space targets [2].

In the mid-course phase, since thrust is not provided to the space targets, the targets are free-flying in high trajectories, typically about 2000 km, with little air to fly over a long distance. In this phase, space targets, debris, and decoys are separated and move together as a result of low air resistance [3]. For a BMD system to intercept the space targets in this phase, it is important to distinguish the space targets from debris and decoys. For this purpose, the HRRP and inverse-synthetic aperture radar (ISAR) images are used [4,5], but it is difficult to expect good performance due to the similar shape of decoys and space targets. Unlike conventional radar signatures such as HRRP and ISAR images, research on identifying targets using micro-Doppler signatures has been extensively studied for decades. Space targets with spin-motor have unique dynamics such as precession, spin, and nutation, whereas decoys and debris have a tumbling or wobbling motion due to being lightweight. These distinct micro-motions lead to distinct micro-Doppler signatures that can be used to classify or identify the space targets from debris and decoys.

In the re-entry phase, the space targets re-enter the atmosphere at a very high velocity. Due to the atmospheric delay, the space targets and others can be largely distinguished, but there is a difficulty in that the flight time is very short.

To classify space targets during the mid-course phase, which is the longest flying time among three phases, early studies have mainly focused on modeling various micro-motions such as precession, nutation, and spin [6,7,8,9,10,11,12]. Each micro-motion is modeled by using a 3D rotation matrix, including Rodrigues’ rotation formula, and is verified from measurements of a simple target model. In addition, cone-shaped space targets and decoys have been modeled by three scattering centers, which are located at the top and both ends of the bottom, to generate micro-Doppler signatures. This method has been widely spread thanks to simple and easy modeling, and it has been also utilized to extract the major scattering centers from the measured micro-Doppler signature. Although the scattering center method is quite effective to model various micro-motions, it can be valid for only simple cone-shaped targets. Even if the shape is slightly complicated, such as a space target with a blunt tip or empennages, it is difficult to apply the scattering center method. In order to extract the scattering point of a complex space target, it is necessary to perform an electromagnetic analysis of various postures over a bandwidth. In addition, it is relatively possible to extract fixed and accurate scattering centers at high frequencies where the target is large compared to the wavelength, but it is difficult to extract dominant scattering centers at frequencies similar to the wavelength of the target. Due to these issues, received signals or radar cross-section (RCS) are often directly obtained through a full-wave electromagnetic simulation according to various postures and incident angles [13,14]. Recently, the concept of shooting and bouncing rays with relative aspect angles has been introduced [13]. In consideration of the symmetrical shape of the space targets, the scattered field according to the angle of incidence on a fixed target is calculated and used to configure a lookup table, and consequently, the scattered field is estimated according to the relative angle of incidence between the target’s orientation and the incident field. This method has the advantage of not only having a shorter calculation time compared to the 3D full-wave electromagnetic simulation but also obtaining a signal similar to the real one considering various scattering mechanisms.

Combining the micro-motions with estimation methods such as the scattering center method, a 3D electromagnetic simulation, or the method of estimating scattered field from a lookup table gives dynamic RCS, which is proportional to the square of a radar received signal, for a certain period of time (dwell time). A spectrogram, one of the micro-Doppler signatures, is transformed from the dynamic RCS by the short-time Fourier transform (STFT), and the cadence velocity diagram (CVD) is also converted from the spectrogram if necessary. The signatures are used to train a classifier, and a large data set is generally required. Therefore, it is essential to obtain an accurate and fast data set.

For a conventional automatic target recognition (ATR) system, features according to micro-motions are extracted from the signature to train a classifier. In [15], a method based on the nonlinear least square and the orthogonal matching pursuit algorithms were introduced to extract the precession frequency and scattering centers. In [8], the micro-Doppler period and micro-Doppler bandwidth were extracted without velocity estimation by using a high-resolution time-frequency transform. Similarly, in [9], the coning, spinning, and appearing frequencies of a cone-shaped object with empennages were extracted by using the correlation figure method and the relationship among three frequencies. Clemente [16] applied the pseudo-Zernike moments to the cadence velocity diagram (CVD) in order to minimize the feature acquisition dependence. Furthermore, Presico [11] presented three feature extraction algorithms—averaging and normalizing the CVD-based, Pseudo-Zernike-based, and Gabor Filter-based vector approaches. In [17], a feature extraction method based on the circular correlation coefficients and the circular average magnitude difference coefficients was introduced.

On the other hand, convolutional neural networks (CNNs) have the advantage that they do not require a feature extraction process because they include not only the feature extraction part but also the classification part. For this reason, since CNNs have been introduced, CNNs have been mainly used as a classifier of the ATR system. Therefore, research on classifying space targets has been changing from extracting high-quality features to designing new neural networks or pre-processing input data. In [18], the transfer learning of AlexNet and SqueezeNet were used to classify the micro-motions based on spectrogram images. In [13], a parallel CNN network that inputs both spectrogram and CVD at the same time has been proposed. In [14], a deep learning network was designed that uses RCS signal patterns rather than micro-Doppler signature images as input data. Recently, Wang [18] has introduced a complex-valued coordinate attention network-based end-to-end recognition method, whose input data are also complex-valued echo data. These CNN models have the advantage of being able to quickly create datasets and train and classify them because the process of transforming echo data to micro-Doppler signatures is not required. However, there is also a disadvantage in that the explainability of the classification result is low. Classification using micro-Doppler signatures has been currently being studied more actively for application to the motion of drones and humans than to application to space targets [19,20,21,22,23,24]. This is because drones and radar technologies have been expanded to the private sector and are attracting attention.

When a BSD system tracks space targets and debris during the mid-course phase, the relative incident angle between the conning axis of a space target and the radar line of sight (RLoS) can be obtained from the geometry of the trajectory of targets and a radar position of the BSD system. So, not only conventional micro-Doppler signatures but also the relative incident angle can be used for the space target classification. In this paper, we generate micro-Doppler signature images considering the relative incident angle and show that the proposed signatures can improve the classification accuracy in a common CNN model.

## 2. Proposed Micro-Doppler Signature Dataset Generation Method

As shown in Figure 1, when a radar tracks space targets and debris during the mid-course phase, the relative angle (θ_r_) between the translation vector ($\hat{v}$) and the RLoS vector ($\hat{n}$) can be easily obtained from a simple vector operation. Therefore, the relative angle can be used as an additional input variable of a deep learning network, and it can also be used in the process of generating a micro-Doppler signature image during the mid-course phase. In this paper, the relative angle is used as the latter approach to maximize the classification accuracy of space targets from debris or decoys. 

**Figure 2:**
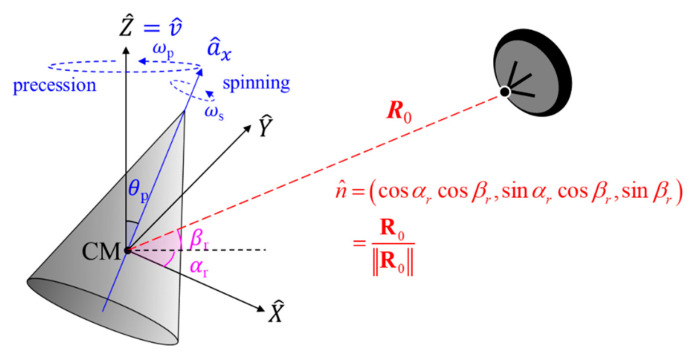
The geometry of a radar and a cone-shaped target with precession and spin rotations.

As is well-known, the space targets with a spin-motor precess so as not to deviate from their trajectory. To model these micro-motions (precession and spinning), the rotation axes should be defined. Figure 2 shows the geometry of a radar and a cone-shaped target with a micro-motion. The origin of the reference coordinate is assumed to be the center of mass (CM) of the target, and **ω**_p_ and **ω**_s_ are the scalar angular velocity for the precession and spinning about the *Z*-axis ($\hat{Z}$) and the target’s longitudinal axis (${\hat{a}}_{x}$), respectively. Here, since the space target translates while precessing, it is assumed that the translation vector ($\hat{v}$) is the same as the precession axis ($\hat{Z}$), and θ_p_ denotes the precession angle which is the angle between the *Z*-axis and the longitudinal axis.

The radar is stationary and located at a distance of *R*_0_ from the target. If the azimuth and elevation angles of the incident field are *α_r_* and *β_r_*, respectively, the unit vector of the RLOS is given by:

(1) $$\hat{n}=\left(\cos\alpha_{r}\cos\beta_{r},\sin\alpha_{r}\cos\beta_{r},\sin\beta_{r}\right)$$

where the relationship between the elevation angle (*β_r_*), and the relative angle (*θ_r_*) is that *β_r_* + *θ_r_* = 90°.

Assuming a high pulse repetition frequency (PRF) and a relatively low angular velocity, the Doppler frequency shift is then approximately:

(2) $$f_{D}=\frac{2f}{c}\left[v+\omega \times r\right]\cdot n$$

where the first term is the Doppler shift due to the object’s translation, and the second term is the micro-Doppler due to the rotation. The first term can be removed by applying various translational motion compensation methods such as the single-scatterer referencing algorithm, multiple-scatterer algorithm, and the phase gradient autofocus technique, while the second term remains after the motion compensation. The micro-Doppler frequency shift is rewritten as:

(3) $$f_{mD}=\frac{2f}{c}\left[\omega \times r\right]\cdot n$$

where **ω** denotes the angular rotation velocity vector, and the object rotates along the unit vector of **ω** with a scalar angular velocity **ω**. On the other hand, **r** is the position vector of a rotating object, and its magnitude is the radius *r* of a circular motion. Therefore, the parenthesis of (3) means the tangential velocity as:

(4) $$v_{⊥}=\omega \times r$$

In addition, the micro-Doppler frequency shift depends on the tangential velocity component in the direction of RLoS from (3). Since the precession axis is assumed to be the *Z*-axis, as shown in Figure 2, the tangential velocity is parallel to the XY-plane. Therefore, the micro-Doppler frequency is maximized when the RLoS is parallel to the XY-plane. Even with the same micro-motion, the micro-Doppler frequency shift depends on the relative angle of the incident field. Conversely, if the relative angle is known, the same micro-Doppler frequency shift can be obtained for the same micro-motion.

**Figure 3:**
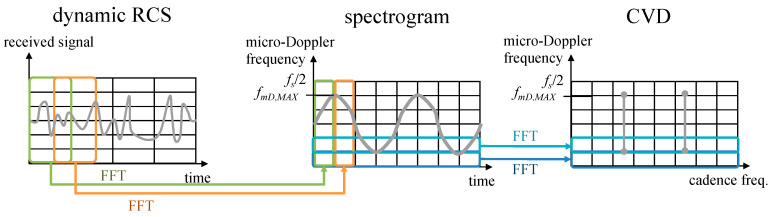
The procedure of transforming a dynamic RCS to the micro-Doppler signature images of the spectrogram and CVD.

Figure 3 shows the conventional procedure of obtaining micro-Doppler signatures images. First, the dynamic RCS, which have complex values, is divided into short periods, multiplied by a window function, and Fourier transform is performed on each chunk. A spectrogram of a two-dimensional image is obtained by performing the fast Fourier transform (FFT) while sliding the window along the time axis. The CVD is obtained by performing an FFT along the time axis for all micro-Doppler frequency bins in the spectrogram. The perpendicular axis of the spectrogram and CVD is the micro-Doppler frequency shift, and the maximum value of the micro-Doppler frequency shifts is half the sampling frequency (*f_s_*). Therefore, the larger the pulse repetition frequency (PRF), the finer micro-Doppler frequency shift can be observed. Meanwhile, the maximum value of the micro-Doppler frequency (*f_mD_*_,*MAX*_) is the maximum value obtained from (3). For example, the maximum micro-Doppler frequency by the precession as shown in Figure 2 is given as follows:

(5) $$f_{mD,MAX}=\frac{2}{\lambda }\omega_{p}r_{p}\cos\beta_{r}$$

Equation (5) means that the micro-Doppler frequency pattern of the spectrogram is compressed in the transverse direction according to the relative incident angle for the precession. Therefore, if the magnitude of the Doppler frequency shift is compensated by the cos*β_r_* term, the same spectrogram image is obtained regardless of the relative angle of the incident field. In CVD, the same signature image can be obtained regardless of the relative angle of the incident field by the same method.

Unfortunately, patterns in a spectrogram are not one-dimensional data, and the micro-Doppler resolution is determined by the ratio of the sampling frequency, *f_s_*, to the number of FFT points. Therefore, even if the image shrunken in the transverse direction by the cos*β_r_* term is stretched again, the image is simply enlarged in the transverse direction due to having the same resolution.

**Figure 4:**
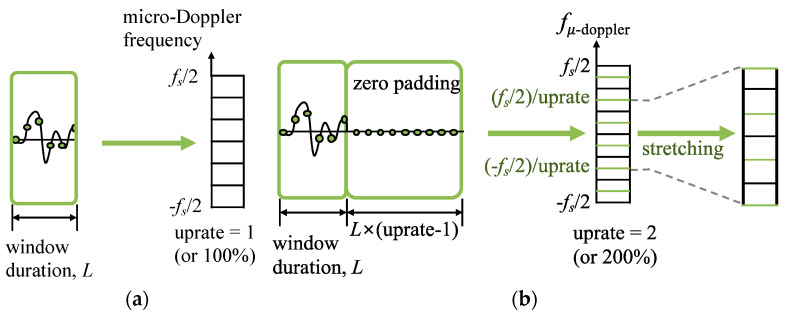
The procedure diagram for zero-padding and signature image stretching for the uprate of (**a**) 1.0 (cos*β_r_* = 1.0) and (**b**) 2.0 (cos*β_r_* = 0.5).

Zero padding does not improve the frequency resolution, but it can have more frequency bins that are more closely spaced in frequency. When calculating the spectrogram, if the spectrogram image is stretched after performing zero-padding as much as the uprate of 1/cos*β_r_*, the micro-Doppler frequency resolution cannot be improved, but it can make it easier to visually resolve the stretched spectrogram pattern from the frequency-domain interpolation. Figure 4 illustrates the procedure of stretching the zero-padded spectrogram image according to the relative incident angle. Here, the uprate is set to 1/cos*β_r_*, and then the zero-padding length and image stretching rate are changed according to the relative incident angle to minimize an unnecessary increase in FFT calculation time.

## 3. Target Models and Dataset Generation

**Figure 5:**
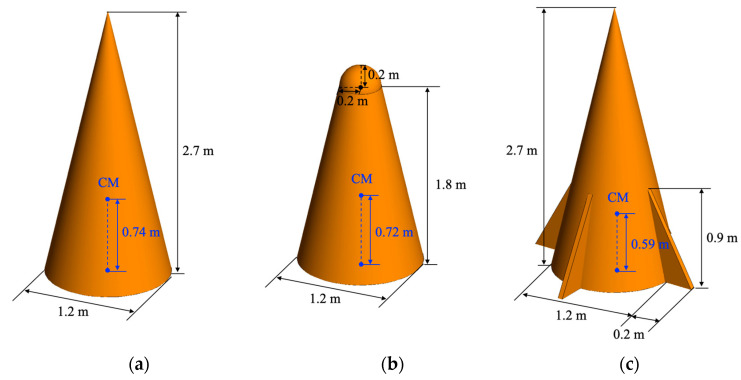

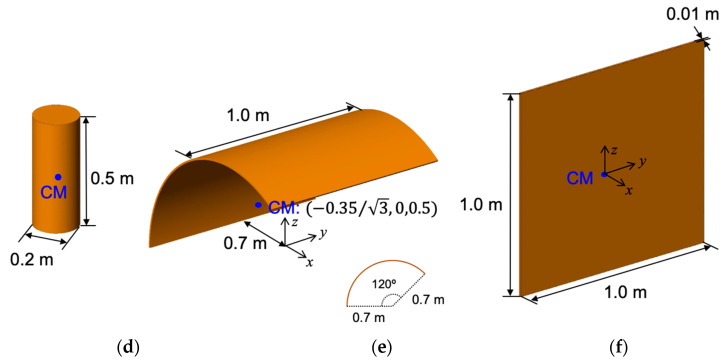
Target object models: (**a**) cone, (**b**) rounded cone, (**c**) cone with empennages, (**d**) cylinder, (**e**) curved plane, and (**f**) squared plane [13].

In order to verify the performance of the proposed micro-Doppler dataset considering the relative incident angle, a dataset is generated for the same target models as the previous study of [13], and the classification performances are compared. Therefore, the same target models as shown in Figure 5 and the same dynamics and radar system parameters summarized in Table 1 are used, so that the dynamic RCS is the same as in the previous work [13], but the spectrogram and CVD images are different. 

**Figure 6:**
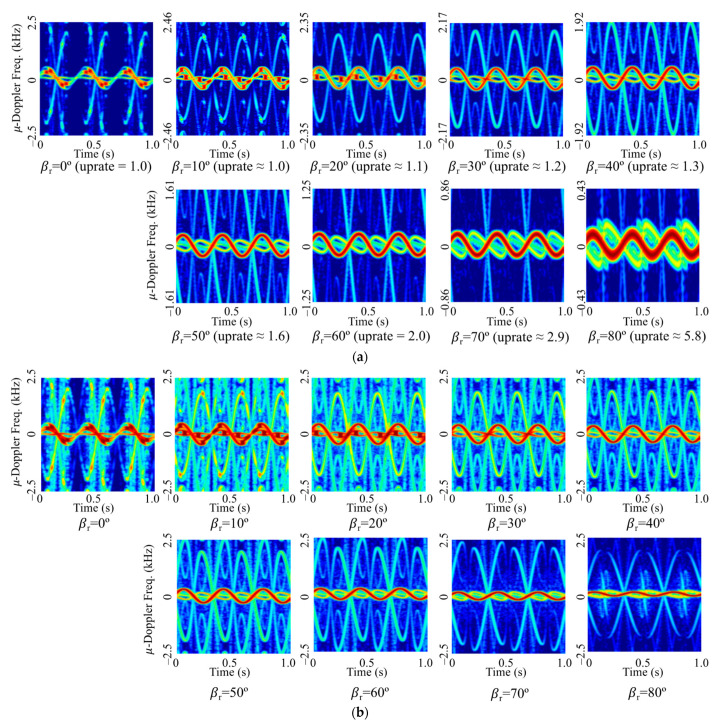
Spectrograms of the rounded cone-shaped model with respect to the relative incident angle (**a**) by using the proposed method and (**b**) conventional method.

Figure 6 shows the spectrogram of the rounded cone for some incident angles when the rotation rate is 3 Hz. For the conventional spectrogram of Figure 6a, the larger the relative angle, the smaller the micro-Doppler frequency shift of the blunt tip. On the other hand, for the spectrogram by the proposed method of Figure 6b, the micro-Doppler frequency shift of the blunt tip is similar regardless of the incident angle. However, since the resolution does not increase even if the uprate is increased as the incident angle increases, the thickness of the micro-Doppler signal line increases and the pattern appears as if it increases. In addition, as the incident angle increases, the micro-Doppler frequency shift by both bottom surfaces increases, unlike the blunt tip, and has a value almost similar to the micro-Doppler frequency shift of the blunt tip. However, the field scattered by the bottom surface is smaller than that by the blunt tip, so the shape of the overall spectrogram is constituted by the blunt tip. Although the magnitude of the micro-Doppler frequency is not the same in the image, an increase in classification accuracy can be expected due to the similarity of the pattern of the blunt tip.

**Figure 7:**
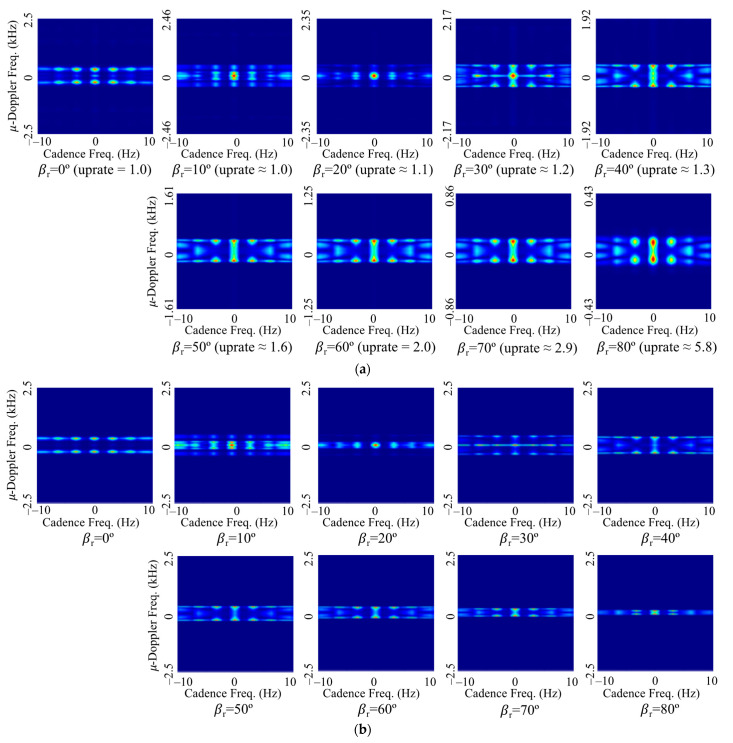
CVD of the rounded cone-shaped model with respect to the relative incident angle (**a**) by using the proposed method and (**b**) conventional method.

For all the CVDs by the rounded cone of Figure 7a, the first peak cadence frequency is located at a certain distance from the middle of the horizontal axis corresponding to the rotation rate of 3 Hz. In particular, as the angle of incidence increases, the micro-Doppler frequency shift decreases, so that the top and bottom of the CVD pattern are compressed. Therefore, although it is suitable for extracting the rotation rate from CVD, it is ineffective for the application to CNN, which classifies based on image patterns. On the other hand, for the CVDs generated by the proposed method of Figure 7b, the maximum value of the micro-Doppler frequency is generated at a similar level, and the CVD patterns at which harmonics are generated as well as the position of the first peak are similar, so it is expected that the classification accuracy will be increased.

To consider the noise effect on the micro-Doppler signatures, the white Gaussian noise is generated and then added to the scattered field so that the signal-to-noise ratio (SNR) varies in the range of −10 dB to 20 dB in 5 dB step, which are identical to the range and step of the previous work [13]. The number of dataset images for each target including both noise-added and noise-free is summarized in Table 2.

## 4. Classification Results Using Deep Learning Network

In this session, we compare the transfer-learning performance of the dataset of micro-Doppler signatures generated by the proposed method in consideration of the incident angle with the performance of conventional signatures. Since the object of our work is to compare performance according to data sets, rather than designing a new deep learning network, AlexNet and ResNet-18, which have shown relatively good performance in the previous study [13], are applied to the transfer learning. The computer used in the experiment was the Intel core CPU i9-10900X 3.70 GHz, 128 GB RAM, and GPU NVIDIA RTX 6000. 

In all experiments, from micro-Doppler signature generation to transfer learning, our work was implemented by using MATLAB. The stochastic gradient descent with momentum (SGDM) was used for the optimizer. The learning rate was 0.001, the maximum number of epochs was 7, and the size of the mini-batch was 128. The generated dataset is randomly divided into three subsets: the training set, the validation set, and the test set. The ratios used are 80% train, 10% validate, and 10% test.

Table 3 shows the classification performance, according to input data and network model. When the conventional spectrogram was used as input data, the AlexNet and ResNet-18 had about 95% and 97%, respectively. When the proposed spectrogram was used as input data, the performance was worse than when the conventional spectrogram was used, and the accuracy was about 92% and 94% for AlexNet and ResNet-18, respectively. That is, the spectrogram to which the proposed method is applied deteriorated about 3% in accuracy compared to the conventional spectrogram.

On the other hand, in the case of the conventional CVD, the transfer learned AlexNet and ResNet-18 have accuracies of about 86% and 88%, respectively. When the proposed CVD images are used as input data, the accuracies are about 90% and 92% for AlexNet and ResNet-18, respectively. That is, it had better classification performance when using the proposed CVD images than when using the conventional CVD images.

**Figure 8:**
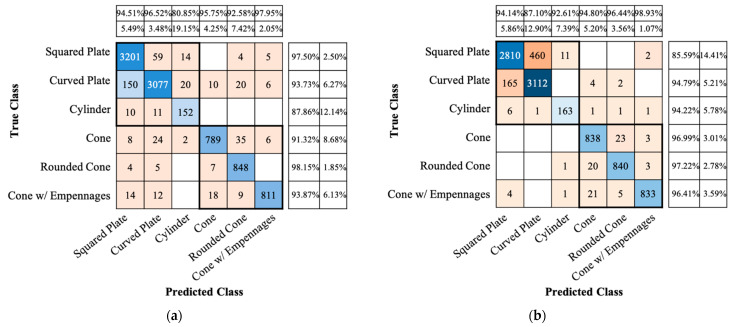
Confusion matrices of accuracy performance of AlexNet transfer-learned using (**a**) the conventional and (**b**) the proposed spectrogram images.

Figure 8 shows the confusion matrices of the test accuracies of AlexNet for the conventional and proposed spectrogram images. The *x*-axis is the true class, and the *y*-axis is the predicted class. Compared the results of the proposed spectrogram images with those of the conventional images, the true positive rates (TPR or recall) of each true class are high or similar, with the exception of 85.59% of the squared plate, and the positive predictive values (PPV or precision) of each predicted class are also high or similar, with the exception of 87.10% of the curved plate. That is, in the case of the squared plate, the result of misclassification as the curved plate was the most, and conversely. The reason is that in the case of the squared and curved plates, there were many similar data in the form of a widespread of micro-Doppler frequency shift in the spectrogram.

**Figure 9:**
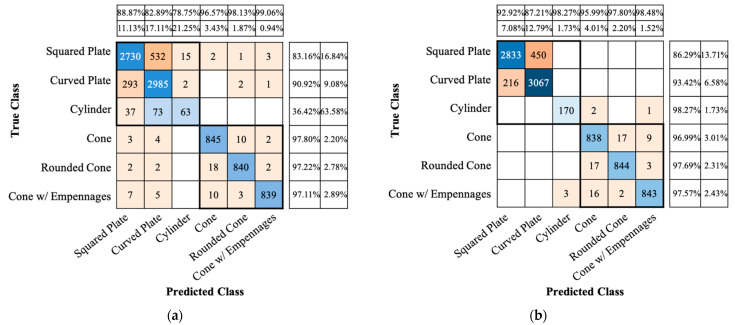
Confusion matrices of accuracy performance of ResNet-18 transfer-learned using (**a**) the conventional and (**b**) the proposed CVD images.

Figure 9 shows the confusion matrices of ResNet-18 for the conventional and proposed CVD images. Similar to the accuracy performance using the conventional CVD images, when the proposed CVD images are used, the squared and curved plates are most often misclassified. However, it can be noticed that both the TPR and PPV of each true and predicted class are similar or higher than the performance of the conventional CVD. That is, it can be noticeable that the proposed CVD increases the classification accuracy.

From the radar point of view, it is important to classify the types of debris and space targets, but it is even more important to classify whether the target is precessing space targets or debris. As shown in Figs. 8 and 9, the proposed micro-doppler signatures are quite effective in improving the classification performance of CNNs to distinguish between tumbling and precession targets, despite more misclassification between curved and spare plates. In particular, the classification performance of the cylinder target is improved because, among tumbling targets, the shape of the cylinder is significantly different from the curved and square plates having four vertices. Therefore, the accuracy performance was recalculated by classifying space targets and debris into two classes instead of six classes and summarized in Table 4. Based on the accuracy performance of classifying into two types of space targets and debris, using CVD rather than spectrogram shows higher classification performance. The proposed micro-Doppler signatures improve the classification accuracies by 0.3–1.1% for both the spectrogram and CVD types. These results are completely opposite to the results in Table 3, and the proposed signatures have superior performance for debris and space target classification. Among the signatures, CVD has a better performance.

## 5. Conclusions

In this paper, we proposed a method to generate a micro-Doppler signature considering the incident angle. Based on the principle that the micro-Doppler frequency shift appears differently depending on the angle between the direction of rotation by precession, which is the main micro-motion of the space targets, and the RLOS, it is proposed to show the constant amount of micro-Doppler frequency shift by the relative incident angle. Micro-Doppler signature data sets were generated for three space target models and three debris models, and transfer learning was performed with AlexNet and ResNet-18. In the case of the proposed CVD, the classification accuracy was increased compared to the case of transfer learning with the conventional CVD, but the classification accuracy decreased in the case of the proposed spectrogram. However, if the classification performance is recalculated into two classes of space targets and debris instead of six classes through the analysis of the confusion matrix, all of the proposed signatures have a classification accuracy of more than 99%. In particular, the proposed CVD showed the highest classification accuracy of 99.94% in ResNet-18. These results are expected to be effectively applied to radar resource management in the classification process of space targets and debris.

## Figures and Tables

**Table 1:** Dynamic and radar system parameters for dataset generation [13].

Parameter	Values	Step
Rotation rate (ω*_p_*)	0.25–3 Hz	0.25 Hz
Incident angle (*β_r_*):	0–85°	5°
Precession angle (*θ_p_*)	4–12°	2°
Initial direction of debris (*θ*_init_)	0–180°	10°
Operating frequency	X-band	
Sampling frequency (or PRF)	5 kHz	Time step: 0.2 ms
Dwell time	1 s	

**Table 2:** The number of generated micro-Doppler signature images [13].

Dwell Time	Signature	Cone	Rounded Cone	Cone w/Empennages	Cylinder	Curved Plate	Squared Plate	Total
1 sec	SP ^1^	8640	8640	8640	1728	32,832	32,832	93,312
	CVD	8640	8640	8640	1728	32,832	32,832	93,312

^1^ SP: spectrogram.

**Table 3:** Validation and Test accuracies of conventional transfer learned CNNs.

CNN Models	AlexNet		ResNet-18	
Type of micro-Doppler signature	SP	CVD	SP	CVD
Previous (val. acc.)	95.40%	86.39%	97.58%	88.66%
(test acc.)	95.15%	86.90%	97.17%	88.97%
Proposed (val. acc.)	91.38%	89.97%	94.03%	91.84%
(test. acc.)	92.12%	90.59%	93.91%	92.11%
Training time	~9 min		~22 min	

**Table 4:** Validation and Test accuracies of conventional transfer learned CNNs.

CNN Models	AlexNet		ResNet-18	
Type of micro-Doppler signature	SP	CVD	SP	CVD
Previous	98.78%	99.45%	99.44%	99.66%
Proposed (val. acc.)	99.82%	99.92%	99.88%	99.94%

## Data Availability

Not applicable.
